# The Interaction between the Gut Microbiome and Bile Acids in Cardiometabolic Diseases

**DOI:** 10.3390/metabo12010065

**Published:** 2022-01-11

**Authors:** Cengiz Callender, Ilias Attaye, Max Nieuwdorp

**Affiliations:** 1 Department of Internal and Vascular Medicine, Amsterdam University Medical Center, 1105 AZ Amsterdam, The Netherlands; i.attaye@amsterdamumc.nl (I.A.); m.nieuwdorp@amsterdamumc.nl (M.N.); 2 Department of Experimental Vascular Medicine, Amsterdam University Medical Center, 1105 AZ Amsterdam, The Netherlands

**Keywords:** gut microbiota, gut-derived metabolites, bile acids, insulin resistance, cardio-metabolic disease

## Abstract

Cardio-metabolic diseases (CMD) are a spectrum of diseases (e.g., type 2 diabetes, atherosclerosis, non-alcohol fatty liver disease (NAFLD), and metabolic syndrome) that are among the leading causes of morbidity and mortality worldwide. It has long been known that bile acids (BA), which are endogenously produced signalling molecules from cholesterol, can affect CMD risk and progression and directly affect the gut microbiome (GM). Moreover, studies focusing on the GM and CMD risk have dramatically increased in the past decade. It has also become clear that the GM can function as a “new” endocrine organ. BA and GM have a complex and interdependent relationship with several CMD pathways. This review aims to provide a comprehensive overview of the interplay between BA metabolism, the GM, and CMD risk and progression.

## 1. Introduction

Cardio-metabolic diseases (CMD) is an umbrella term covering a spectrum of diseases (e.g., metabolic syndrome, type 2 diabetes, and atherosclerosis), with increasing prevalence, morbidity, and mortality worldwide [1,2]. While current treatments can diminish the damage caused by the complications of these illnesses, more effective strategies to prevent disease development and alter the course of disease progression (for example, by reducing risk factors, such as obesity, dyslipidemia, and insulin resistance) are needed [3]. Thus, research into the etiologic pathways that drive CMD is urgently needed. In this regard, the gut microbiome (GM) is a scientific field of particular interest.

The GM is a complex ecosystem of 100 trillion (10^14^) bacteria, archaea, fungi, protozoa, and viruses [4]. The function of these microorganisms extends the ability to extract nutrients from otherwise indigestible food [5]. Additionally, the GM can modulate host energy expenditure, immunity, and cardio-metabolism through the production of bioactive molecules [3,6,7]. These metabolites range from short-chain fatty acids (SCFA) such as butyrate, propionate, and acetate, which are often associated with positive metabolic outcomes, to trimethylamine N-Oxide (TMAO) and Imidazole Propionate (ImP), which are negatively associated with metabolic health [8,9]. 

Finally, the GM is involved in the modulation of endogenous compounds, in particular, that of bile acids (BAs). These intriguing molecules play a vital role in the digestion of food and have long been known to function as signalling molecules implicated in relevant pathways for CMD [10]. In this review, we discuss the interplay between GM and CMD in humans, with a particular focus on current insights into the three-way interaction between GM, BAs, and human metabolism. 

## 2. The Gut Microbiome

Many internal and external factors regulate the gut microbiome composition and function. Internal factors include the host’s genetic makeup and age and external factors such as: geographical location, diet, and medication use (external factors) [11]. To what extent these known factors explain the vast differences in global GM diversity remains a topic of debate [12,13]. 

Recently, ethnicity has also been recognised as another factor of the gut microbiome composition [13,14]. The specific causes of these ethnic differences are not yet known. Both genetic and cultural factors likely contribute to this diversity. The first studies into the consequences of these different GM profiles on the prevalence and severity of CMD have recently been published, specifically finding that the alpha diversity of non-Caucasian inhabitants of Amsterdam is lower than that of Caucasians. Cohort studies are ongoing to gain more insight into these findings [13,15] 

The GM can influence CMD risk via various bioactive metabolites, discussed below. Briefly, SCFA, TMAO, and ImP are known gut-derived metabolites influenced by dietary factors and associated with CMD [8,9,16,17]. 

A multitude of confounding factors complicate the interpretation of the role of the GM in human disease. It is challenging, for example, to deduce whether a particular composition of the GM or specific microbial function is a cause or a consequence of disease. Individual differences in microbiome composition are significant (within healthy and in disease populations) [18,19]. A generalisable definition of a health- or disease-associated microbiome or characteristics thereof is complicated to establish. 

In addition to confounders in study populations, findings on the role of the gut microbiome in CMD can be challenging to reproduce between research groups because of various analytical methods being used to determine the gut microbiome composition (e.g., 16 S rRNA gene sequencing versus shotgun metagenomic sequencing) and post-sequencing bioinformatics approaches (e.g., differences in bioinformatics pipelines) [20].

Despite these challenges in human gut microbiome research, the GM has been shown to influence several seemingly unrelated fields, from shaping the human immune system [21,22], activating the gut–brain axis, for example, by modulating the hypothalamic satiety response [23], and influencing the cardiovascular event rate [24]. What has reproducibly been shown is that higher diversity in gut bacterial species is often associated with a healthy phenotype [25,26]. 

## 3. An Overview of the Gut Microbiome in CMD Perspective

The GM has been associated with multiple diseases that fit within the CMD spectrum [25]. Many of these studies are associative by nature, but several pre-clinical and clinical trials have been performed that highlight the importance of the GM in CMD [27,28,29]. 

Considering the associations between gut microbiome compositional differences and CMD, influencing the complete microbial composition in the gut could effectively reduce the risk of morbidity and mortality driven by CMDs. Pioneering work in rodent models points towards a causal correlation between the GM and a lean or an obese phenotype [30,31,32]. These studies explored the effects of the GM in mice with an obese phenotype and lean phenotype, performing a faecal microbiota transplantation (FMT) from obese to lean. This caused an obese phenotype to develop in formerly lean mice. It seemed to have this effect through changing energy homeostasis; more energy was extracted from the same diet in (formerly) lean mice after the obese GM transplantation. Another mice study showed the effectiveness of the drug resveratrol in improving glucose homeostasis through GM mediation [33].

In humans, causal evidence is more limited. Transplanting a healthy donor an allogenic GM through a FMT has been shown to improve metabolic markers [28,34,35,36,37]. For example, transplanting a healthy donor (allogenic) GM was demonstrated to limit the progression of type 1 diabetes after 12 months [27].

A prospective study showed that FMT could increase insulin sensitivity in patients with metabolic syndrome [28]. These hallmark studies imply that the GM is causally involved in CMD and suggest that studying the potential of interventions that alter the GM to target CMD holds merit.

Non-alcoholic fatty liver disease (NAFLD) is another disease categorised in the CMD spectrum. NAFLD is a highly prevalent condition characterised by increased hepatic fat storage and inflammation. It can progress to non-alcoholic steatotic hepatitis (NASH), cirrhosis, and hepatocellular carcinoma [38], in increasing the order of severity of the disease. The incidence of this spectrum of diseases is rising, and it is expected to continue to do so in the coming years. Furthermore, it is one of the leading (and growing) indications for liver transplantations [39]. In recent years, it has been established that diet and the GM play an essential role in the progression of NAFLD [40,41]. Significant advances have been made linking the (gut-derived) metabolome to NAFLD and NASH. This link has also been found through the interaction of the immune system and the gut microbiota and is reviewed elsewhere [42,43,44,45].

A recent review examined the current state of FMT, which outlines promising possibilities to improve the effectiveness of the procedure [46]. One way might be to find microbes with metabolic pathways that provide key metabolites that help ameliorate disease—for example, by introducing SCFA-producing bacteria in obese individuals [16,28]. Alternatively, one could replace the GM with an allogenic microbiota that produces fewer damaging metabolites. 

One crucial area of ambiguity in this field is the definition of a “healthy” bacterial microbiota. The lack of precise characteristics limits the scope of meta-analyses [47]. Multiple initiatives such as the Human Microbiome Project have been started to gain more insight into the composition and function of a “good” versus a “bad” microbiome [48]. In this regard, (international) reference values for microbiota diversity and beneficial strain quantities have not been established yet.

Specific bacterial taxa have often been implicated in disease states; however, the complexity of bacterial taxonomy and the limitation of current genomic analyses techniques show that these associations are not always reproducible [49]. The majority of the studies that investigated the relationship of the GM in CMD have used 16 S rRNA gene sequencing, which is a crude method that mainly gives insight into bacterial composition but does not directly provide insight into gene activity or metabolic function [20]. More robust methods are emerging, using shotgun metagenomic sequencing techniques to better understand the GM’s functionality, not only the composition [50]. However, one parameter validated in multiple cohort and intervention studies is gut microbial alpha diversity [51,52].

One crucial concept with much ongoing research is the subject of bacterial translocation. The concept of bacterial translocation was postulated more than a decade ago to contribute to the low-grade chronic inflammation observed in CMD [53,54]. Yet, except for certain infectious and pathological conditions, gut bacteria are generally confined to the intestine and strictly separated from the circulation. Although the immune system’s local (intestinal) interaction with bacteria still contributes to inflammatory tone in CMD, the current dogma states that bacterial components such as lipopolysaccharides [55] and flagellin [56] translocate into the system and activate the immune system in several tissues. In addition, gut bacteria are a critical phase between dietary input and the regulation of human metabolism via the production of microbial metabolites. Current estimates suggest that around 15% of all plasma metabolites are derived from gut bacteria [57]. Although some bacterial metabolites and their roles in CMD have been unravelled in great detail (discussed below), the majority of microbial metabolites, their link with diet, and their putative role in human metabolism are yet to be revealed. 

The overall consensus in the field exists that a higher gut microbial alpha diversity is associated with better (CMD) health outcomes. Therefore, influencing the complete microbial composition in the gut could be an effective and novel method to reduce morbidity and mortality due to CMD. Strategies to control the microbial alpha diversity include antibiotic interventions, dietary interventions, and interventions using pre-, pro-, or syn-biotics [11].

## 4. Gut Microbiome-Derived Metabolites

As mentioned above, the GM can also produce several GM-derived metabolites linked with CMD in both rodent and human studies. Many of these metabolites, such as SCFA, TMAO, and ImP, are produced via nutritional (dietary) resources. In the intestines, SCFA are a product of the bacterial fermentation of dietary fibre (resulting in mainly propionate, butyrate, and acetate), but can also be produced from protein sources by the GM [58]. SCFA have been well studied and positively affects insulin resistance, hypertension, obesity, and atherosclerosis [16,59,60]. However, oral supplementation of butyrate did not improve insulin sensitivity in obese metabolic syndrome subjects [61], whereas oral propionate could improve insulin resistance in these subjects [62].

TMAO and ImP are metabolites produced via the GM, where dietary red meat is a major substrate for their production. Both metabolites have been linked to insulin resistance in rodent and human studies [8,9,17]. Moreover, TMAO has been linked to atherosclerosis and multiple conditions that span the domain of CMD. Therefore, it is likely that dietary interventions that alter the substrates for the production of these metabolites via the GM can have important clinical effects. This has been shown by a recent randomised clinical trial with a crossover design, which showed that plasma TMAO levels were higher in subjects eating an animal-based diet compared to a plant-based diet. A disturbed lipid profile also accompanied these findings in the animal-based diet group [63]. 

Another interesting hypothesis that has been recently published is that dietary fructose, when eaten in significant amounts, can reach the colonic gut microbiota where the GM metabolises them to toxic metabolites (e.g., glycerate instead of acetate) [64]. Thus, reducing fructose consumption or improving the colonic catabolism of dietary fructose by altering the GM composition can reduce NAFLD. 

Microbial compositions associated with a diseased state are often termed “dysbiotic” or “dysbiosis”. This controversial term indicates that the gut microbial composition is altered and functionally different from a healthy state. However, whether dysbiosis is a cause or a consequence of the disrupted metabolic state remains controversial [47]. Several factors drive CMD dysbiosis, including medication usage, ethnicity, insulin resistance, obesity, smoking, and socioeconomic factors [48,65]. In addition to these factors, endogenous molecules, including BAs, alter the gut microbiome. 

Since this review will focus on BAs, we will first summarise BA metabolism and the BA-mediated signalling pathways implicated in CMD. 

## 5. Bile Acid Metabolism 

BAs are amphipathic molecules excreted into the duodenum after a meal to aid the emulsification and absorption of dietary lipids, fat-soluble vitamins, and cholesterol. Their central role in fat absorption has historically implicated BAs in metabolic dysregulation, including metabolic syndrome, type 2 diabetes, and NAFLD. BAs also facilitate the elimination of cholesterol and bilirubin that undergo colonic conversion by gut bacteria. In addition to their role in the digestion of lipids and as signalling molecules, BAs have been shown to have a bactericidal effect through their detergent properties. They lower the overall quantity of intestinal bacteria (favouring bacteria resistant to BAs), change the relative abundance of different bacterial phyla, and promote microbial diversity [66,67,68,69]. 

The liver synthesises BA from cholesterol in a multistep process, synthesising cholic acid and chenodeoxycholic acid. These so-called primary BAs are then conjugated with the amino-acids taurine or glycine, making them more hydrophilic and less cytotoxic. Importantly, in contrast to humans, rodents can produce ursocholic acid and muricholic acids, particularly beta-muricholic acids. Significant differences in human and rodent BA metabolism present challenges in terms of translating the experimental rodent findings to humans. Therefore, it is essential to consider these limitations as most of the studies linking the GM with BA metabolism in CMD are performed in rodents [70].

Bile salts tightly regulate their synthesis via negative feedback inhibition by activating the nuclear receptor farnesoid-X receptor (FXR) Activated hepatic FXR reduces the expression of CYP7A1 via the nuclear orphan receptor small heterodimer partner (SHP). The activation of FXR in the distal ileum induces the expression of FGF15, which is subsequently released into the portal bloodstream. The secreted FGF15 binds the FGFR4/β-klotho receptor complex expressed on hepatocytes. This induces the activation of various signal transduction routes, including the extracellular signal-regulated kinase/c-Jun N-terminal kinase (ERK/JNK) pathway, which reduces the activity of CYP7A1. Although FXR is considered to be the principal regulator of bile salt synthesis, other pathways are involved in the negative feedback of bile salt synthesis, including intestinal TGR5 (Takeda G protein-coupled receptor 5) signalling [71,72].

**Figure 1:**
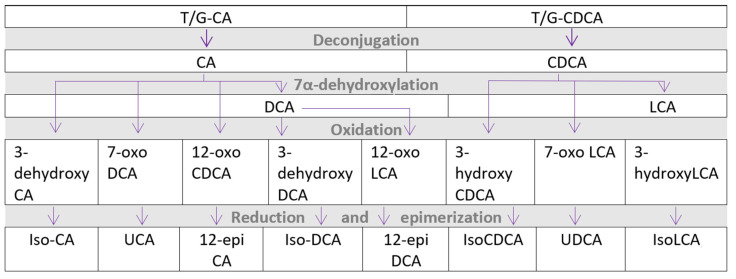
Microbial metabolism of bile acids to secondary and tertiary bile acids. CA—cholic acid, CDCA—chenodeoxycholic acid, DCA—deoxycholic acid, LCA—lithocholic acid, T/G—conjugated with taurine or glycine, UCA—ursocholic acid, and UDCA—ursodeoxycholic acid.

**Figure 2:**
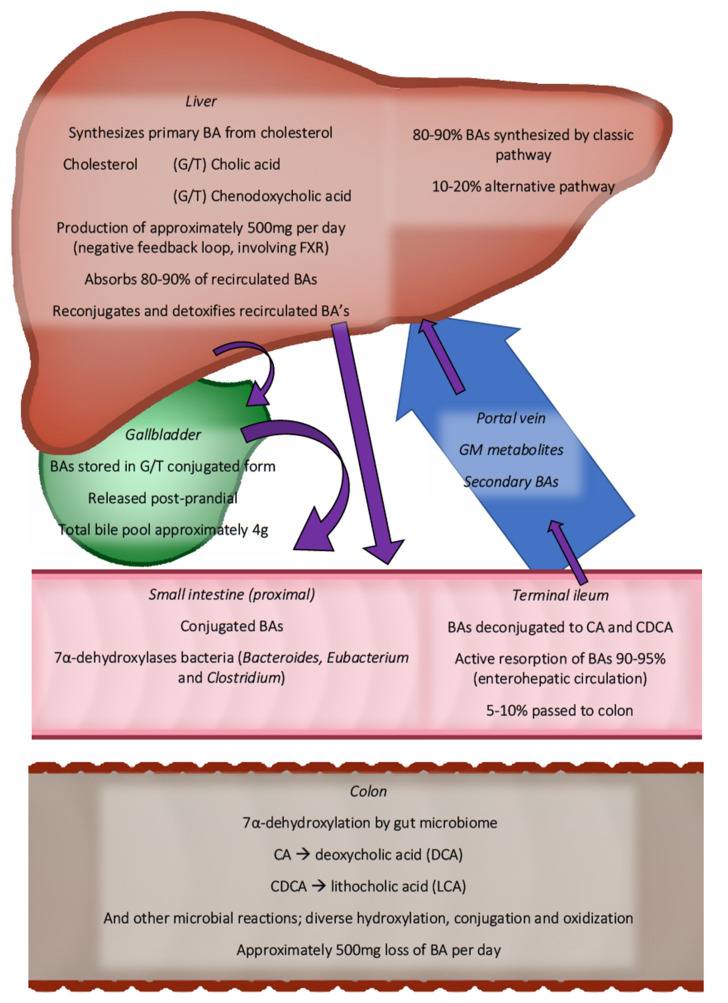
Overview of enterohepatic circulation gut microbiota in bile acid in human metabolism. Purple arrows show the route of bile acids collectively. Only the most abundant bile acid species are shown. BAs are synthesised in the liver (500 mg daily) and are transported through the bile ducts to the small intestine. Starting in the distal ileum, microbiota are found, increasing in density towards the distal colon. More than 90% of bile acids are reabsorbed in the distal ileum. The remainder continues, where they are transformed into secondary bile acids. In total, 500 mg of BAs are lost daily with the stool. BA—bile acid, CA—cholic acid, CDCA—chenodeoxycholic acid, FXR—farnesoid-X-receptor, and G/T—glycine or Taurine conjugated.

Most intestinal BAs are reabsorbed by the distal intestine, with 90–95% of BAs entering the enterohepatic circulation. The remainder enters the colon, where the GM converts them to secondary and tertiary BAs (see Figure 1) [73]. The microbial conversion makes the BAs more hydrophobic. BAs activate several receptors, including TGR5, pregnane-X-receptor, FXR, and vitamin D receptor, affecting the metabolic pathways of relevance for CMD [74]. TGR5 and FXR, in particular, have a strong affinity for hydrophobic BAs (see Figure 2 for an overview of bile acid circulation).

## 6. FXR and TGR5 Pathway

As mentioned above, FXR is a receptor that, when activated, inhibits the formation of BA in the liver. The activation of FXR in the intestine by BA leads to the activation of fibroblast growth factor 15 (FGF15) in rodents (equivalent to FGF19 in humans), leading to the downregulation of the CYP7A1 gene and thereby less BA synthesis. However, studies show contradictory findings, in which both the activation and blocking of FXR show positive effects on CMD outcomes [75]. One possible explanation for this may lie in the fact that FXR can be activated as well as inhibited by BA [76]. Another receptor that BAs activate is TGR5, which is expressed in multiple tissues [71]. The activation of TGR5 leads to several cascades of events that ultimately affect energy expenditure and glucose homeostasis (via activation of glucagon-like peptide-1 (GLP-1)). TGR5 activation also stimulates the conversion of inactive to active thyroxine, which affects whole-body metabolism [77]. The role of BAs in TGR5-mediated glucose metabolism has also been shown in rodent studies, where the administration of BAs to obese mice improves glucose homeostasis. However, their effect remains elusive in human metabolism. Mainly secondary BAs affect TGR5 receptors, and these BAs can also be formed through the microbiota. Therefore, modulating the gut microbiota can alter energy expenditure, glucose levels, and whole-body metabolism via TGR5 receptor activation. This has also been shown to be the case in a clinical study investigating the beneficial effects of a gastric sleeve operation regarding the gut microbiota, BA composition, and glucose levels [78]. After the gastric sleeve procedure, endogenous BA cholic acid-7-sulfate in the gut increased. This BA is a TGR5 agonist and induces glucagon-like-peptide 1. This increased insulin sensitivity in both mice and humans. See Table 1 for an overview of the current associations of BAs and CMD.

The majority of the studies investigating BA’s role in metabolic health have used rodent models, which have a vastly different BA composition than humans [70]. Mice, for example, have almost no glycine-conjugated Bas and have native ursocholic acid and muricholic acids. These Bas have other signalling actions than the human BA varieties. 

Clinical studies focusing on FXR and TGR5 have shown interesting results. For example, FXR- and TGR5-agonist obeticholic acid was used in human trials, showing a positive effect on plasma lipids. However, it also had dose-dependent side effects [89], the most common of which was intolerable pruritus. More worrisome is the fact that obeticholic acid worsened the serum lipid profile and increased the development of liver failure in primary biliary cholangitis patients with cirrhosis. These side effects might be mitigated by selectively targeting BA receptors in the intestines. Such drugs are currently in development [74,89]. The long-term consequences are unknown, precluding the widespread and safe use of such medications. Therefore, new clinical studies are urgently needed to put these findings into perspective and eventual therapeutic use.

## 7. Cardio-Metabolic Disease, Gut Microbiome, and Bile Acid Interplay

As mentioned before, multiple cardio-metabolic diseases (e.g., type 2 diabetes, non-alcoholic fatty liver disease, and atherosclerosis) are characterised by a gut microbial dysbiosis [90]. However, the interaction between BAs and dysbiosis in these conditions has been poorly studied, especially clinical studies. One landmark randomized-controlled, double-blind study [91] found that the most commonly used anti-diabetic drug, metformin, alters the gut microbiota composition and affects the plasma BA levels of treatment-naïve type 2 diabetes subjects. Metformin changed the gut microbiota by increasing the abundance of *Akkermansia muciniphila (A. muciniphila).* Moreover, this study also found that the plasma levels of BAs were raised in the metformin-treated group but not the placebo group. This finding was likely caused by the increased abundance of gut microbiota-produced enzymes that can accelerate the deconjugation of conjugated BAs.

However, clinical studies investigating the interaction between gut microbiota dysbiosis, BA metabolism, and atherosclerosis are still lacking, whereas rodent studies have been performed with interesting results. One study in ApoE-/-, C57BL/6 mice showed that dietary resveratrol (a polyphenol) could alter atherosclerosis risk and progression [92]. Resveratrol altered the gut microbiota composition by increasing the levels of *Bifidobacterium* and *Lactobacillus*. These bacteria can increase the levels of unconjugated BAs from conjugated BAs. Moreover, resveratrol lowered the levels of TMAO by reducing trimethylamine production, which ultimately affected atherosclerosis formation and progression [93]. In this regard, the GM has been found to have a direct signalling effect in atherosclerosis development through the production of TMAO [9,94]. Gut bacteria can catabolise choline and L-carnitine into TMAO, known to drive atherosclerosis, as germ-free mice fed L-carnitine or choline did not have increased atherosclerosis [95]. Additionally, TMAO has been linked to hypertension. In several studies, an association between gut dysbiosis (through decreased alpha diversity) and hypertension has been found [96,97,98,99,100]. Animal studies have proven it can influence blood pressure through FMT and antibiotics [101,102]. Human studies did not have sufficient power to find causal links between GM metabolites and hypertension. In the 2020 review by Vallinou et al. [103], possible associations are summarised. 

Finally, studies are urgently warranted into the three-way interaction between NAFLD, the gut microbiota, and BA signalling in cardio-metabolism. NAFLD increases in prevalence and can lead to hepatitis and cirrhosis, with high morbidity and mortality rates [104]. One observational study in humans found that the BA profile, mainly the primary conjugated BA, was associated with fibrosis in subjects suffering from non-alcohol steatohepatitis (NASH) [80]. Interestingly, these findings were also correlated with an increase in *Bacteroides* and *Lactobacilli* strains in the microbiome. These included strains with the potential to deconjugate BAs. 

Another study in mice showed that lower concentrations of secondary BAs lead to lower activation of FXR, which eventually leads to higher serum levels of triglycerides and glucose [105,106]. In contrast, the first donor faecal transplantation study showed improved liver histology and gene expression in NAFLD-NASH subjects [107], underscoring the therapeutic applicability of GM in this disease. 

A human intervention study further supported this. In it, a beneficial strain, *E. hallii*, was supplemented. Indeed, a single-dose administration of this *E. hallii* (recently renamed *Anaerobutyricum soehngenii*) improved glucose metabolism via BA composition in human CMD patients [108]. 

## 8. Conclusions and Future Perspectives

With their interconnected nature, BA and GM are a fascinating field of study. The complex connections between BA metabolism, FXR signalling, and dyslipidemia could be promising targets for novel interventions, diminishing morbidity and mortality in CMD. Theoretically, changing the GM and BA signalling could be an easy target, as dietary and pharmaceutical interventions are relatively easy to implement. 

We are still far from using these relatively new targets for CMD treatment. Multiple reasons exist for this. The primary reason is that the current research is made up associative studies rather than research managing to prove causal relations. Is the observed dysbiosis an alteration in BA signalling in a diseased condition of CMD the cause of the CMD, a consequence of it, or both? 

It is essential to realise that the vast majority of papers on the role of the gut microbiome in CMD are focused on bacteria. However, GM comprises more than bacteria (i.e., it includes the genomes of fungi, yeast, viruses, and bacteriophages). Historically, these microorganisms have often been associated with poor metabolic health and infections [109]. However, in the context of the GM, it becomes clear that a complex relationship between the microorganisms exists, which can help produce beneficial and harmful metabolites [3]. Therefore, much more research is needed to better understand these kingdoms in the context of the holobiont and the eventual implications and possible strategies to improve human (metabolic) health [110]. It is beyond the scope of this review to address other gut microbiome members. Still, we would like to emphasise that insight into the interrelation between bacteria and other gut community members is likely to be of great importance for human CMD development and the optimisation of microbiota-targeted interventions. 

To answer these questions, the field needs to evolve beyond association and rodent studies and realise clinical trials and large-scale population-based prospective cohort studies. Ideally, these extensive cohort studies should be multi-ethnic as ethnicity has been shown to play an important role in gut microbial profiles [12,13]. Moreover, microbial sequencing techniques should focus more on shotgun metagenomic sequencing to better understand the functional profile of the microbial community, rather than being stuck at the compositional level [111]. This is becoming more realistic as shotgun metagenomic sequencing and mass spectrometry have become more affordable. By combining well-organized clinical trials with (multi-ethnic) large-scale prospective cohort studies, the field can move from association to causation and disentangle the complex interplay between the gut microbiota, BAs, and cardio-metabolic diseases. 

## Figures and Tables

**Table 1:** In vivo bile acids and associated CMD in the literature. Specific bile acids are only shown if associations with CMD were found in clinical or animal studies. There are some contradictory results due to different physiologies in mice and humans. Only relevant studies are shown.

Bile Acids	Source	Associated Disease	References
Total bile pool		After vertical sleeve gastrectomy in mice, bile acid pool is increased. This caused more weight loss and improved glucose tolerance through a farnesoid-X-receptor-mediated pathway.	[79] Mice
		Liver fibrosis: Higher concentration	[80] Human
Primary bile acids	Synthesised in liver	Obesity: Increased concentration, intestinal FGF levels decreased.	[81] Human
		Type 2 diabetes: Taurine-conjugated BAs are increased.	[82] Human
		NASH: Less concentrated, lowering TGR5 activation.	[83] Mice
Chendeoxycholic acid	Primary	NAFLD: increased concentration.	[84] Human
Secondary bile acid pool	Microbial metabolites, synthesised by (among others) *Bacteroides*, *Bifidobacterium*, *Clostridium*, *Eubacterium*, *Lactobacillus*, *Listeria*, *and* *Ruminococcus*	Agonists of FXR and other nuclear receptors. Increased expression of TGR5. Increased insulin sensitivity.	[85,86,87] Both human and mice studies
Lithocholic acid	Secondary BA	Cytotoxic in unsulphated form. Liver fibrosis: high concentrations.	[80,88] Human

## Glossary

ASCVD	Atherosclerotic cardiovascular disease
BA	Bile Acids
CMD	Cardio-metabolic disease
CVD	Cardiovascular disease
FXR	Farnesoid-X receptor
GIM	Gastro-intestinal microbiome
Holobiont	Superorganism of host and commensal microorganisms
LXR	Liver X receptor
Prebiotics	Food supplements or components that stimulate the health of the gut ecosystem
Probiotics	Living microorganisms believed to be beneficial to human health when ingested
SCFA	Short-chain fatty acid
Synbiotics	Food supplements containing both pre- and probiotics
TGR5	Takeda G protein-coupled receptor 5
TMAO	Trimethylamine N-oxide
